# Assessing Knowledge, Preventive Practices, and Depression among Chinese University Students in Korea and China during the COVID-19 Pandemic: An Online Cross-Sectional Study

**DOI:** 10.3390/healthcare9040433

**Published:** 2021-04-08

**Authors:** Bo Zhao, Fanlei Kong, Eun Woo Nam

**Affiliations:** 1 Department of Health Administration, Graduate School, Yonsei University, 1 Yonseidae-gil, Wonju 26493, Korea; zhaobo@yonsei.ac.kr; 2 Centre for Health Management and Policy Research, School of Public Health, Cheeloo College of Medicine, Shandong University, Jinan 250012, China; 3 NHC Key Lab of Health Economics and Policy Research, Shandong University, Jinan 250012, China; 4 Health City Research Center, Institute of Health and Welfare, Yonsei University, 1 Yonseidae-gil, Wonju 26493, Korea

**Keywords:** COVID-19, Chinese university students, China, Korea, preventive practices, depression

## Abstract

To investigate the knowledge, preventive practices, and depression of Chinese university students living in South Korea and Mainland China during the COVID-19 outbreak and explore the determinants of depression among these students, an online cross-sectional questionnaire survey was conducted from 23 March to 12 April 2020. The online questionnaire included questions on knowledge and preventive practices related to COVID-19, and the Patient Health Questionnaire-9 was used to diagnose depressive symptoms. A total of 420 Chinese university students were finally included in the study (171 students from South Korea and 249 students from Mainland China). The majority of these students had a good level of knowledge of COVID-19. Students living in South Korea displayed better preventive practices than those living in Mainland China; however, the proportion of students (28.7%) with moderate-to-severe depression in this group was relatively higher than that (18.9%) of the Mainland Group (χ^2^ = 5.50, *p* < 0.05). More severe depression was related to high levels of concern about family members and contracting COVID-19 as well as suspecting themselves of having come into contact with patients. Displaying more preventive behaviors decreased the depressive symptoms in both groups. These data could be used as a reference for further studies in different regions to take measures (e.g., psychological counseling and encouragement for physical activities) to reduce depressive symptoms in university students.

## 1. Introduction

An increasing number of infectious diseases have recently led to serious economic and social consequences globally [1,2]. These emergent public-health events not only result in physical pain but also have a profound psychological impact [3,4], such as inducing panic, anxiety, and depression (see the examples of the Middle East Respiratory Syndrome [5] and Ebola virus disease [6] outbreaks). Links between anxiety/depression and viral diseases such as influenza A and other influenza viruses have also been proven [7]. Further, psychological stress may lead to immune dysfunction, which has a negative impact on human health [8]. For instance, the SARS virus caused a series of psychological problems such as post-traumatic stress disorder in patients [9]. Therefore, it is necessary to determine the population’s mental health status during a health emergency as early as possible as well as make recommendations and provide interventions.

The coronavirus disease 2019 (COVID-19) first broke out in Wuhan, Hubei Province, China, in December 2019 and had been found in 188 countries and regions worldwide as of 20 May 2020 [10]. On 30 January 2020, the World Health Organization (WHO) declared it a worldwide pandemic [11]. The disease is contagious, widespread, and has no known drugs to target it [12,13]. The increasing numbers of confirmed patients, suspected cases, and provinces affected by the outbreak made Chinese people feel worried and scared [14,15]. Coupled with the ongoing social distancing and isolation measures implemented in several countries and regions, this outbreak has led to additional mental health problems such as stress, anxiety, depressive symptoms, insomnia, and fear globally [16,17,18]. The World Health Organization has also noted that mental health and psychological well-being in different target groups must be considered during the COVID-19 outbreak [19].

As college students are transitioning to adulthood, this is a crucial time in their personal development [20]. They may not only have to face stress related to their academic performance but also tackle adult-like responsibilities without having yet achieved the skills and cognitive maturity of adulthood [21]. For example, research has found that some college students lack the experience to handle emergencies, lack analytical and forecasting skills, and display impulsive behavior or a vulnerable and unstable mood [22,23]. Moreover, several researchers have proposed that mental health problems are becoming increasingly common among college students [24,25,26], which can be tackled by receiving clear and consistent information [27]. Potential problems such as anxiety, stress, and depression can negatively affect academic performance [28,29]. 

Students in China and South Korea were directly or indirectly affected because the COVID-19 outbreak coincided with the Chinese lunar New Year holiday and opening of South Korean universities. To prevent the outbreak from escalating, universities in China and South Korea postponed the beginning of the semester in the first half of 2020; canceled all campus events such as workshops, conferences, sports, and other activities [30]; and changed to an online learning model [31]. Nonetheless, the mental health of college students forced to stay at home for a long time because of fewer collective activities may be affected, leading to anxiety and depressive symptoms [32,33]. Thus, the psychological condition of university students cannot be overlooked and must be monitored.

Since the viral outbreak was first reported in China, the Chinese have been targeted and blamed for the spread of COVID-19. An example of this is the use of the terms “China virus” and “Wuhan virus” by the media [34]. Chinese students account for the largest proportion of foreign students studying in South Korea [35]. Given their remoteness from their interpersonal networks and low contact with relatives and friends, international students have to rely on themselves when studying abroad [36]; they often live alone or with one other person in a room or dormitory [37]. Moreover, these young adults have never known or experienced a severe disease outbreak in China before. Previous research shows that studying abroad adds another layer of stress that can exacerbate pre-existing mental problems [38]. Hence, there is a high probability that loneliness abroad along with global discrimination and stress over the pandemic may affect their academic performance and mood [39].

The risk and protective factors contributing to the depression and anxiety of university students during the COVID-19 pandemic have been well researched. A study of university students in the United Arab Emirates and Jordan demonstrated that adequate knowledge, good attitudes, and low-risk practices can protect them against contracting COVID-19 [40,41]. Moreover, having relatives and acquaintances infected with COVID-19 is a risk factor for increasing anxiety among university students [42,43]. In their study of Nigerian university students, Rakhmanov and Dane found that an increase in knowledge may help decrease anxiety levels [44]. In addition, a study of French university students showed that knowledge of the pandemic may reduce its negative impact (e.g., stress and anxiety) in the vulnerable populations [45]. Even when anticipatory coping behaviors are active, many people remain afraid of distressing situations and their awareness of coping strategies is poor [46]. Young adults practicing social distancing and undertaking low levels of activities have poorer mental health [47]. Hence, although some university students have a good level of knowledge about COVID-19 and its preventive practices, health authorities still should take their depressive status seriously [48]. However, no research has thus far examined the extent to which knowledge of COVID-19 and the risk factors leading to depression during the pandemic affect Chinese university students domestically and overseas [49]. Therefore, using an online questionnaire survey, this study was aimed at exploring the conditions and determinants of knowledge, preventive practices, and depression among Chinese university students in Mainland China and South Korea during the COVID-19 pandemic.

## 2. Materials and Methods

### 2.1. Participants

An online cross-sectional survey was used to collect data from respondents through an anonymous questionnaire. We conducted the online survey from 23 March to 12 April 2020 (According to the World Health Organization, as of 31 March 2020, there were 82,545 confirmed cases in China (total deaths: 3314) and 9786 confirmed cases in South Korea (total deaths: 162)) during the COVID-19 pandemic and 420 university students were finally included in the analysis. Of these, 171 were Chinese university students studying in South Korea (referred to as the International Group hereafter) and 249 were studying in Mainland China (Mainland Group).

### 2.2. Procedure

First, the English version of the COVID-19 questionnaire used by Wang and colleagues [16] was adopted. Second, the sample size needed for the study was calculated using the G*Power 3.19 program. Based on the parameters of a two-sided test and χ^2^ test, a residual variance of 0.83, α probability = 0.05, and power = 0.95 for F tests and linear multiple regression analysis, the minimum total sample size was estimated to be 356.

Because of the limited accessibility to respondents owing to the social distancing policy during the COVID-19 outbreak, we conducted an online cross-sectional survey. With the help of collaborators and native speakers, a Chinese version of the questionnaire written in simplified Chinese characters was created and placed on survey platforms (Naver Form Tool in South Korea, Surveystar in Mainland China). Before conducting the survey, we revised and verified the content of the questionnaire through an online pilot survey among several students to ensure that the statements were appropriate and understandable. Native researchers in both countries checked its readability, comprehension, and convenience for the respondents to answer.

Potential respondents were sent a link to participate in the questionnaire. During the initial screening, a statement of the purpose of the research and assurance of the confidentiality and privacy of individuals was placed on the first page of the survey questionnaire. Participants could only complete the questionnaire after reading this statement and clicking “AGREE” to confirm their consent. Hence, we ensured that consent forms had been completed and participants had been informed about the questionnaire before the survey was conducted. All participants were told that they had the right to stop the survey at any time. In addition, we stipulated that the main questions in the survey were mandatory questions (as shown below in Section 2.4). In other words, participants had to answer the questions before they submitted the online questionnaire. Any question left blank made the final submission of the online questionnaire impossible. All these measures resulted in a 100% response rate for our study.

In total, 461 respondents were recruited via snowball sampling, wherein we recruited further respondents among their acquaintances. There were 180 responses from Chinese students in South Korea collected from 23 March to 8 April 2020 and 281 responses from Chinese students in Mainland China collected from 2 to 12 April 2020 (461 in total). To ensure that we only surveyed university students, 41 respondents who answered employed, unemployed, or other to the occupation question were removed, leaving 420 students (171 in the International Group and 249 in the Mainland Group). All respondents expressed their willingness to participate and understood the background and purpose of the study.

### 2.3. Measurements

The questionnaire (see Supplementary File) used in our study included two sections: (1) a questionnaire on COVID-19 and (2) the Patient Health Questionnaire-9 (PHQ-9). First, the questionnaire on COVID-19 consisted of questions that covered (1) demographic and physical health data, (2) knowledge and perceptions of COVID-19, and (3) preventive practices against COVID-19 in the past 14 days. Most questions in this questionnaire show reasonable validity and reliability according to Wang et al.’s research on the Chinese population [16]. The Yonsei Global Health Center changed Part E (preventive measures) and Part F (additional information) according to the specific situations in the two countries. Second, the PHQ-9, which has been validated for use in primary care [50], was adopted to provide a baseline for the incidence of depression. After data collection, we compared the knowledge and preventive practices of COVID-19 and depression among Chinese university students in South Korea and China.

### 2.4. Description of the Variables

#### 2.4.1. General Demographics

In this study, to reflect the demographic characteristics of the respondents, the basic survey asked questions related to respondents’ sex, age, education level, marital status, family size, whether they had medical insurance, whether they had chronic diseases, whether they had traveled abroad in the past 14 days, and whether they had experienced quarantine. The choices for educational level were undergraduate and graduate and the choices for marriage status were single and married. In terms of family size, the choices were 1-person family, 2-person family, 3–5 persons family, and more than a 6-member family. Questions about having medical insurance, chronic illnesses, traveled abroad, and self-quarantined were answerable by either yes or no. Respondents were also asked about their self-assessed physical condition, which was answerable by either good or above and fair or below.

#### 2.4.2. Knowledge and Perception of COVID-19

Knowledge and perception of COVID-19 and other topics were evaluated, which included questions on the student’s knowledge of transmission pathways, satisfaction with the information, sources of related information, confidence about being diagnosed, degree of concern about this disease, perceived probability of becoming infected, and concern about family members.

#### 2.4.3. Preventive Practices of COVID-19

Nine basic preventive practices were incorporated into the questionnaire. The responses to these questions corresponded to the degree to which a measure was practiced on a daily basis (1 = never do this and 5 = do this every day). The total score indicated how well preventive practices were performed. The reliability (Cronbach’s α) of the preventive practices of the COVID-19 scale in this study was 0.72 and the validity was 0.78.

#### 2.4.4. Patient Health Questionnaire-9

Depressive symptoms were diagnosed based on the nine criteria for depression in the Diagnostic and Statistical Manual of Mental Disorders published by the American Psychiatric Association [51]. Each question on the PHQ-9 was answered as follows (with their corresponding score): not at all (0 points), several days (1 point), more than half the days (2 points), and almost every day (3 points) [52]. Respondents were divided into five groups according to their total score (0–4, 5–9, 10–14, 15–19, and 20–27), which corresponded to minimal or none, mild, moderate, moderately severe, and severe depression, respectively [53]. The higher the score, the more severe the depression.

With a sensitivity of 88% and a specificity of 88% for detecting major depressive disorders, a score of 10 is recommended as the cut-off for diagnosing depression [54]. Thus, in this study, respondents who scored 10 or more points were classified as moderate-to-severe, and respondents who scored fewer than 10 points were classified as “minimal-to-mild”. The reliability and validity of the PHQ-9 were 0.89 and 0.90, respectively.

### 2.5. Statistical Analysis

In this study, STATA 15.0 (Stata Corp., College Station, TX, USA.) and SPSS 24.0 (IBM Corp., Armonk, NY, USA) were used to conduct the statistical analysis as follows. First, descriptive statistics, *t*-tests, and chi-squared tests were performed to compare each variable between the International Group and Mainland Group. Second, to explore the determinants of the different depression levels, hierarchical regression was performed.

## 3. Results

### 3.1. General Characteristics

As shown in Table 1, there were significant differences between the two groups in terms of age, education level, marital status, family size, medical insurance, and self-quarantine. The mean age of the International Group was 24.08 ± 4.14 years compared with 22.12 ± 2.28 years in the Mainland Group. There were higher proportions of graduate students, married respondents, and respondents with families comprising one to two members in the International Group than in the Mainland Group. In addition, a higher percentage of students did not have medical insurance (16.4%) in the International Group. By contrast, nearly half (47.8%) of the Mainland Group had experienced being self-quarantined. In total, more than 90% of respondents reported a good self-assessed physical condition.

### 3.2. Knowledge of COVID-19

Table 2 shows the students’ responses about their knowledge of COVID-19. About 99.8% knew that the virus could spread through droplets, 88.8% knew that it could be transmitted via contact with contaminated objects, and 66.9% knew that it could be transmitted through air. The two groups were also up-to-date on information on infections, deaths and recoveries. Regarding sources of information, 92.1% of respondents obtained information from one to three sources such as the Internet, TV, and family members.

Between the International Group and Mainland Group, there were significant differences in terms of confidence about being diagnosed, concerns about the disease, and high perceived probability of becoming infected. Although relatively more respondents in the Mainland Group were highly confident about being diagnosed and highly concerned about the disease, more respondents in the International Group had a high perceived probability of infection. Among International Group students, 34.5% thought they were highly likely to be infected (19.3% in the Mainland Group). However, some also thought they were more likely to survive after infection (91.8% in the International Group vs. 86.7% in the Mainland Group). Furthermore, no statistically significant difference was found in terms of concern about family members.

### 3.3. Differences in Preventive Practices between the Groups

Table 3 shows the performance of preventive measures against COVID-19 in the International Group and Mainland Group. Overall, the two groups were significantly different in terms of seven practices. The mean scores of the International Group were significantly higher than those of the Mainland Group in five practices: covering mouth when coughing and sneezing; washing hands with soap and water; washing hands immediately after coughing, rubbing nose, or sneezing; washing hands after touching contaminated objects; and avoiding public transportation. On the contrary, the Mainland Group achieved higher levels of performance than that of the International Group in terms of the sitting in one row while having a meal and avoiding meeting more than 10 people.

### 3.4. Depressive Symptoms Resulting from the Analysis

A chi-squared test was performed to determine the relationship between the level of depressive symptoms and memberships of the International Group and Mainland Group. Depressive symptoms were categorized into minimal-to-mild and moderate-to-severe (cut-off score of 10) in this study. As Table 4 shows, the difference between the two groups was confirmed as statistically significant (χ^2^ = 5.50, *p* < 0.05); the depression status of the International Group was more severe than that of the Mainland Group.

A simple linear stepwise regression analysis was conducted to explore the factors affecting the depression status of respondents, including all the variables. The results in Table 5 show that satisfaction with the information, patients’ contact history, concern about family members, and self-assessed physical condition were statistically significantly related to respondents’ depression. These four variables were subjected to hierarchical regression in different models in combination with variables such as demographic characteristics, knowledge score, and preventive score.

Table 6 displays the results of the hierarchical regression analysis of the determinants of depression. In Models 1 and 2, there were no statistically significant relationships between age, sex, education level, or marital status and the depression scores of respondents. Students who had a better assessment of their health had lower depression scores. As more variables were added into Models 3 and 4, the factors patients’ contact history, highly concerned about family members, and highly concerned about the disease were associated with statistically significant increases in depression scores. In the four models, the preventive practice scores all had significantly negative relationships with depression scores. In short, the better the performance of preventive practices by students, the lower the depression score. The Durbin–Watson value was close to 2, indicating that the observed value was independent. Although the R^2^ value was low, the p values of the F test in the four models were all less than 0.01, showing a strong correlation of the interpretative power of the models.

## 4. Discussion

### 4.1. Knowledge and Preventive Practices of International Group and Mainland Group Students

This study found that our sample university students had a certain degree of knowledge of COVID-19, concurring with other research results [16,55]. Combined with the findings on satisfaction with the information and information sources, most of them correctly understood how the virus is transmitted and have received detailed information on cases. This shows that the publicity work and health education of schools, health institutions, and the mass media are having an impact [56]. Although a high proportion of students perceived themselves as having high confidence about being diagnosed and that they would survive after infection, many reported being highly concerned about family members in this study. This is also consistent with the fact that as confirmed and suspected cases continue to increase, more provinces and countries are affected by the pandemic [14], which increases public attention.

The effects of the COVID-19 outbreak on Chinese university students’ psychological state and their associated factors have been studied [16,32,48]; however, these studies have only included respondents from Mainland China. After the case of Patient No. 31 (a confirmed case who participated in a gathering in Daegu at the Shincheonji Church of Jesus), a sudden outbreak in South Korea attracted worldwide attention and suggested that upgraded quarantine and isolation measures were necessary [57,58]. Because most International Group students in South Korea live alone, their fears are exacerbated by the fact that they may have to experience a prolonged quarantine period by themselves (83.1% had self-quarantined). This may also explain why students in both groups experienced similar concerns about this disease and family members. In addition, a number of students from the Mainland Group perceived a high probability of contracting the disease. This is also consistent with the situation in Mainland China, where COVID-19 has spread throughout almost every province since January 2020 [59]. Overall, both groups did well in performing preventive practices. The Mainland Group only performed better than the International Group in terms of avoiding using elevators and sitting in one row while having a meal. Therefore, effective measures to prevent the virus should be continued, such as propagandizing through health education and publicity work by health institutions and the mass media in Mainland China.

### 4.2. Depression Status of the International Group and Mainland Group Students

Depressive disorders are one of the most common mental disorders, with a lifetime prevalence of 6.9% and a 12-month prevalence of 3.6% in the Chinese population [60]. The average PHQ-9 scores in these two groups were 7.20 (95% CI: 6.390–7.800) for the International Group and 6.20 (95% CI: 5.583–6.819) for the Mainland Group, and the proportion (28.7%) of the International Group who experienced mild-to-severe symptoms was much higher than that of the Mainland Group (18.9%). Therefore, the prevalence of depression in the college students who participated in this study (12.6%) was higher than average [61]. Factors such as performing preventive practices, patients’ contact history, concern about family members, and concern about the disease and their relationships with the depression scores of these respondents also showed that the COVID-19 outbreak may impact the psychological state of university students, especially International Group students. Fearing that COVID-19 would have psychological consequences similar to other infectious diseases [7,9], universities in South Korea need to provide the necessary psychological interventions and health education measures for these students continuously.

Improving university students’ knowledge of the virus and related preventive practices by providing clear and consistent information during this pandemic improves their psychological health [27]. A lack of knowledge of COVID-19 may cause students to be excessively worried about the damage brought on by the pandemic, resulting in a higher risk perception and more feelings of panic and anxiety [62]. As demonstrated in this study, the more comprehensive the preventive measures, the better the psychological state of university students, the lower their risk of mild depression, and the more positive their response to the pandemic.

The thoughts and feelings of university students could also affect their mental health. This study showed that students who felt good about their bodies and did not suspect themselves as having come into contact with a patient, those with lower levels of stress, and those who were not as worried about their families and the disease had lower depression scores. During this outbreak, the influence of rumors cannot be overlooked [63]. Negative and false information on the pandemic may result in great psychological consequences for students because this may make them feel negative and require the companionship of family and friends at this time [64], which may not be possible for those studying abroad because of bans on transportation and migration. Thus, social and school support provided by governments and universities is still needed to help them develop and maintain a positive mindset.

Another point about the positive relationship between physical activities and mental health is that social distancing and working at home are expected to play a role to a greater or lesser degree in the short to medium term. Even light physical exercise could help relieve some of the negative psychological health impacts among isolated older adults due to COVID-19 [65,66]. To promote physical activity, public health initiatives should target particular populations (e.g., men and young adults) that are more vulnerable to the harmful impacts of physical distance and/or self-isolation [67].

In this battle of pandemic prevention and control, various coping strategies have been developed for university students by the governments in China and South Korea. In China, local governments and academic institutions emphasize the importance of preventive measures (e.g., controlling movement and wearing masks) and the need for mental health support (e.g., psychological consultation and aid)—and not only for young adults [68]. In South Korea, to help students cope with the mental pressure, university authorities have organized programs such as online experience-sharing competitions and encouraged students by offering rewards and financial aid. The required food and healthcare materials are supplied to ensure the safety of international students who need to quarantine [69]. In addition, activities and postings on the online communities of Chinese international students have had a positive effect on health information sharing and social support [70].

### 4.3. Implications

Considerable effort and support by the universities of the two countries should be ongoing to help students thrive in this pandemic. Governments and local authorities should be conscious of the significance of providing adequate guidance and protection to international students in such uncertain and frightening times. The mental health of Chinese university students in both China and South Korea needs to be further ascertained to elucidate the psychological impact in different regions due to the COVID-19 pandemic.

### 4.4. Limitations and Future Research

This study had several limitations. Owing to the restrictions on activities brought on by the COVID-19 pandemic, several adjustments had to be made. First, a web-based questionnaire was adopted in this study, which may have some shortcomings. One example is that self-assessed levels of physical condition and depression scores may not always be aligned with the real situation, because respondents may choose a socially desirable response. Second, the snowball sampling method was employed; therefore, it may not be possible to make statistical inferences from the sample that are applicable to the population since respondents were not randomly selected. Third, the importance and effect of beliefs on depression have been emphasized by many studies. Given that the study populations in the two countries in this research are both young Chinese university students (i.e., ethnically homogeneous), the relationship between beliefs and depressive status was not analyzed. Fourth, the severity of the pandemic varies by region; thus, the effects of the pandemic on depression among university students may also differ by region. Therefore, it is necessary to conduct further studies of their depression status, and we are planning to conduct a prospective study on a comparable group. Notwithstanding the above limitations, this study provides useful information on knowledge, preventive practices, and depressive symptoms among Chinese students in two countries (China and South Korea), which had suffered the largest COVID-19 outbreaks at the time this study was conducted. Our results could be used as an evidence-based reference in the provision of psychological interventions for university students in different areas during the outbreak.

## 5. Conclusions

The present study investigated the knowledge, preventive practices, and depression symptoms of Chinese university students living in South Korea and Mainland China during the COVID-19 outbreak as well as explored the determinants of depression among both groups. The results showed that the majority of respondents had a satisfactory level of knowledge of COVID-19. International Group students displayed better preventive practice than Mainland Group students; however, the percentage of students with moderate-to-severe depression was higher in the International Group. Depression was associated with high levels of concern about family members and about contacting COVID-19, and worry about having had contact with patients. Performing preventive behaviors may be associated with a decrease in the depressive state in both groups. As an initial study of the impact of COVID-19 on Chinese university students in two countries, these data could be used as a reference for further studies in different regions to take measures (e.g., psychological counseling and encouragement for physical activities) to reduce depressive symptoms in university students.

## Figures and Tables

**Table 1:** Demographics and general characteristics of the respondents.

Variables	International Group (*n* = 171) *n* (%)	Mainland Group (*n* = 249) *n* (%)	Total (*n* = 420) *n* (%)	*t*/χ^2^ (Pearson)	*p*
Sex					
Male	57 (33.33)	76 (30.52)	133 (31.67)	0.370	0.543
Female	114 (66.67)	173 (69.48)	287 (68.33)		
Age					
Mean ± S.D.	24.08 ± 4.14	22.12 ± 2.28	22.90 ± 3.30	5.427 ^a^	<0.001
Education Level					
Undergraduate	98 (57.31)	177 (71.08)	275 (65.48)	8.509	0.004
Graduate	73 (42.69)	72 (28.92)	145 (34.52)		
Marital Status					
Single	159 (92.98)	242 (97.19)	401 (95.48)	4.153	0.042
Married	12 (7.02)	7 (2.81)	19 (4.52)		
Family Size					
1 member	9 (5.26)	1 (0.40)	10 (2.38)	32.126	<0.001
2 members	24 (14.04)	8 (3.21)	32 (7.62)		
3–5 members	134 (78.36)	221 (88.76)	355 (84.52)		
6 members or more	4 (2.34)	19 (7.63)	23 (5.48)		
Medical insurance					
No	28 (16.37)	16 (6.43)	44 (10.48)	10.699	0.001
Yes	143 (83.63)	233 (93.57)	376 (89.52)		
Chronic illness					
No	159 (92.98)	232 (93.17)	391 (93.10)	0.006	0.940
Yes	12 (7.02)	17 (6.83)	29 (6.90)		
Traveled abroad					
No	166 (97.08)	244 (97.99)	410 (97.62)	0.366	0.545
Yes	5 (2.92)	5 (2.01)	10 (2.38)		
Self-quarantined					
No	29 (16.96)	119 (47.79)	148 (35.24)	42.230	<0.001
Yes	142 (83.04)	130 (52.21)	272 (64.76)		
Self-assessed physical condition					
Good or above	153 (89.47)	227 (91.16)	380 (90.48)	1.206	0.752
Fair or below	18 (10.53)	22 (8.84)	40 (9.52)		

Note: International Group refers to Chinese college students studying in South Korea and Mainland Group refers to Chinese college students studying in Mainland China. ^a^: *t*-test.

**Table 2:** Differences in knowledge of COVID-19.

Variables	International Group (*n* = 171) *n* (%)	Mainland Group (*n* = 249) *n* (%)	Total (*n* = 420) *n* (%)	*t*/χ^2^ (Pearson)	*p*
Route of transmission					
Droplets (agree)	171 (100)	248 (99.60)	419 (99.76)	0.688	0.407
Objects (agree)	145 (84.80)	228 (91.57)	373 (88.81)	6.213	0.045
Air (agree)	116 (67.84)	165 (66.27)	281 (66.90)	1.037	0.595
Updated information					
Infections (yes)	168 (98.25)	249 (100)	417 (99.29)	4.400	0.036
Deaths (yes)	169 (98.93)	247 (99.20)	416 (99.05)	0.144	0.704
Recoveries (yes)	163 (95.32)	245 (98.39)	408 (97.14)	3.447	0.063
Number of information sources					
1–3	161 (94.15)	226 (90.76)	387 (92.14)	1.608	0.205
4–6	10 (5.85)	23 (9.24)	33 (7.86)		
Satisfaction with the information					
Satisfied	157 (91.81)	239 (95.98)	396 (94.3)	3.274	0.070
Dissatisfied	14 (8.19)	10 (4.02)	24 (5.7)		
Confidence about being diagnosed					
High	101 (59.06)	192 (77.11)	293 (69.76)	15.647	<0.001
Low	70 (40.93)	57 (22.89)	127 (30.24)		
Concern about this disease					
High	123 (61.93)	155 (62.25)	278 (66.19)	4.246	0.039
Low	48 (38.07)	94 (37.75)	142 (33.81)		
Perceived probability					
Become infected (high)	59 (34.50)	48 (19.28)	107 (25.48)	12.379	<0.001
Survive after infection (high)	157 (91.81)	216 (86.74)	373 (88.81)	2.618	0.106
Concern about family members					
High	139 (81.29)	192 (77.11)	331 (78.81)	1.060	0.303
Low	32 (18.71)	57 (22.89)	89 (21.19)		
Knowledge score					
Mean ± S.D	13.95 ± 1.88	13.99 ± 1.92	13.97 ± 1.90	−0.184 ^a^	0.854

Note: ^a^: *t*-test.

**Table 3:** Preventive practices taken against COVID-19 by Chinese university students in Mainland China and South Korea. (Mean ± S.D).

Variables (Score: 1–5)	International Group (*n* = 171)	Mainland Group (*n* = 249)	Total (*n* = 420)	t
1. Wearing mask regardless of the presence or absence of symptoms	4.32 ± 0.79	4.27 ± 0.89	4.30 ± 0.86	0.52
2. Covering mouth when coughing and sneezing	4.67 ± 0.80	4.36 ± 1.02	4.49 ± 0.95	3.28 ***
3. Washing hands with soap and water	4.84 ± 0.44	4.62 ± 0.66	4.71 ± 0.59	2.83 ***
4. Washing hands immediately after coughing, rubbing nose, or sneezing	4.18 ± 1.05	3.98 ± 1.13	4.06 ± 1.10	1.76 *
5. Washing hands after touching contaminated objects	4.93 ± 0.38	4.47 ± 0.78	4.66 ± 0.69	7.11 ***
6. Avoiding public transportation	4.73 ± 0.66	4.58 ± 0.72	4.65 ± 0.70	2.23 *
7. Avoiding elevators	3.44 ± 1.40	4.18 ± 1.19	3.88 ± 1.33	−5.81 ***
8. Sitting in one row while having a meal	2.80 ± 1.70	4.22 ± 1.18	3.64 ± 1.57	−10.13 ***
9. Avoiding meeting more than 10 people	4.74 ± 0.89	4.76 ± 0.62	4.75 ± 0.74	−0.22

Note: * *p* < 0.05; *** *p* < 0.001.

**Table 4:** Difference in depressive symptoms between the students in Mainland China and South Korea.

PHQ-9	International Group (*n* = 171) *n* (%)	Mainland Group (*n* = 249) *n* (%)	Total (*n* = 420) *n* (%)	
Total score ^a^	7.20 ± 0.41	6.20 ± 0.31	6.60 ± 5.14	
Minimal-to-mild ^b^	122 (71.35)	202 (81.12)	324 (77.14)	χ^2^ = 5.50 *
Moderate-to-severe ^b^	49 (28.65)	47 (18.88)	96 (22.86)	

Note: ^a^: the total score of the PHQ-9 is mean ± SD; ^b^: cut-off score of 10 points; * *p* < 0.05.

**Table 5:** Stepwise regression analysis of the factors of depression due to COVID-19.

Dependent Variable	Independent Variables	β	S.E.	β’	*t*	*p*	[95% C.I.]	
PHQ-9 Scores	Constant	13.793	3.186		4.329	0.000	7.501	20.084
	Concern on family members	1.069	0.386	0.205	2.772	0.006	0.308	1.890
	Patients’ contact history	0.574	0.231	0.185	2.489	0.014	0.119	1.030
	Satisfaction with the information	−1.351	0.671	−0.148	−2.013	0.046	−2.676	−0.026
	Self-assessed physical condition	−1.491	0.594	−0.179	−2.509	0.050	−2.388	−0.002

Note: S.E. = Standardized Error; C.I. = Confidence Interval.

**Table 6:** Hierarchical regression analysis of the determinants of depression using the PHQ-9 (*n* = 420).

PHQ-9								
Variables	Model 1		Model 2		Model 3		Model 4	
	β	*t*(*p*)	β	*t*(*p*)	β	*t*(*p*)	β	*t*(*p*)
Constant	12.122	3.439 ***	13.137	3.713 ***	11.586	3.296 ***	10.264	2.936 **
Preventive practice score	−0.165	−3.221 ***	−0.155	−3.027 **	−0.139	−2.745 **	−0.138	−2.749 **
Age	0.199	1.691	0.214	1.823	0.213	1.842	0.225	1.966 *
Sex								
Male	(ref)		(ref)		(ref)		(ref)	
Female	−0.145	−0.547	−0.097	−0.178	−0.020	−0.037	−0.231	−0.431
Educational level								
Undergraduate	(ref)		(ref)		(ref)		(ref)	
Graduate	−0.401	−0.531	−0.466	−0.620	−0.609	−0.820	−0.706	−0.963
Marital status								
Single	(ref)		(ref)		(ref)		(ref)	
Married	−0.990	−0.0742	−1.186	−0.892	−0.938	−0.714	−0.898	−0.693
Knowledge belief score	−0.142	−1.025	−0.149	−1.081	−0.109	−0.836	−0.165	−1.279
Satisfaction with the information								
Low	(ref)		(ref)		(ref)		(ref)	
High	−1.517	−1.415	−1.486	−1.392	−1.316	−1.249	−1.486	−1.426
Self-assessed physical condition								
Fair or below			(ref)		(ref)		(ref)	
Good or above			−1.865	−2.208 *	−1.702	−2.040 *	−1.501	−1.820
Patients’ contact history								
No					(ref)		(ref)	
Yes					−2.842	−0.983	−2.900	−1.015
Not sure					4.413	3.539 ***	4.069	3.287 ***
Concern about family members								
Low							(ref)	
High							1.580	2.624 **
Concern about this disease								
Low							(ref)	
High							1.098	2.081 *
F	2.778 **		3.063 **		3.875 ***		4.456 ***	
R^2^	0.045		0.056		0.087		0.116	
Adjusted R^2^	0.029		0.038		0.064		0.090	
Durbin–Watson	1.972							

Note: * *p* < 0.05; ** *p* < 0.01; *** *p* < 0.001.

## Data Availability

The questionnaire used in the study and datasets can be made available upon request from the corresponding author.

## Supplementary Materials

The following are available online at https://www.mdpi.com/article/10.3390/healthcare9040433/s1, Study Questionnaire.
