# Supplementary material for: Assessing Knowledge, Preventive Practices, and Depression among Chinese University Students in Korea and China during the COVID-19 Pandemic: An Online Cross-Sectional Study
Source: Healthcare (Basel). 2021 Apr 8;9(4):433. doi: 10.3390/healthcare9040433 (PMC8067962; doi:10.3390/healthcare9040433)
Supplement: Supplementary file 1 [file healthcare-09-00433-s001.pdf]

Study Questionnaire

Date of Survey (dd/mm/yyyy): ___________

Questionnaire on COVID-19

Thank you very much for your willingness to participate in a multinational study entitled "Immediate psychological responses and associated factors during the initial stage of the 2019 novel coronavirus (2019-nCoV) epidemic among the college students." The entire survey will take about 20 minutes to complete. No personally identifiable information will be collected.

Part 1

Part A: Demographics

1. Gender: ◯ Male ◯ Female
2. Age: ___________
3. Education attainment
   - ◯ University: Bachelor
   - ◯ University: Master or PhD
4. Residential area during the COVID-19 outbreak
   Please specify City (County) ________
5. Marital status
   - ◯ Single
   - ◯ Married
6. Employment status
   - ◯ Student,
   - ◯ Employed
   - ◯ Unemployed
   - ◯ Others
7. Parental status?
   - ◯ Not applicable
   - ◯ No children

◯ Has child 16 years or under

◯ Has child older than 16 years

8. Household size:

◯ 1 person

◯ 2 persons

◯ 3-5 persons

◯ 6 persons or more

9. Have you traveled outside of your residential country in the past 14 days?

◯ No

◯ Yes, please specify visited countries ___________

Part B: Symptoms and physical health status

1. Symptoms of body discomfort in the past 14 days (please check all that apply)

☐ Persistent fever (>38°C for at least 1 day)

☐ Chills

☐ Headaches

☐ Myalgia

☐ Cough

☐ Difficulty breathing

☐ Dizziness

☐ Coryza

☐ Sore throat

☐ Persistent fever and cough or difficulty breathing

☐ Nausea, vomiting, diarrhoea

2. Did you see a doctor in the clinic in the past 14 days?

◯ No (skip to #6)

◯ Yes

3. Were you admitted to the hospital in the past 14 days?

◯ No

◯ Yes

4. Were you tested for COVID-19 / 2019-novel coronavirus in the past 14 days?

◯ No

◯ Yes

5. Were you under quarantine by health authority in the past 14 days?

◯ No

◯ Yes

6. Please self-rate your current health status

◯ Very good

◯ Good

○ Fair
○ Poor
○ Very poor

7. Do you have medical insurance from private sector?
   ○ Yes
   ○ No
8. Do you suffer from a chronic illness diagnosed by physician?
   ○ No
   ○ Yes, please specify _______________
9. Do you have experiences self-isolation?
   ○ Yes
   ○ No

Part C: Contact history

1. Have you directly or indirectly contacted patients suffering from COVID-19?
   ○ No (skip to Part D)
   ○ Yes
   ○ [Don't know]
2. Extent of direct and indirect contact history of COVID-19 patients (please check all that apply)
   ☐ Close contact with a confirmed case
   ☐ Indirect contact with a confirmed case ("contact of direct contact")
   ☐ Contact with a suspected case
   ☐ Contact with infected materials
   ☐ [Don't know]

Part D: Knowledge and belief about COVID-19

1. Does the COVID-19 transmit through…	Agree	Disagree	Don't know
a. Droplets	○	○	○
b. Contact via contaminated objects	○	○	○
c. Airborne	○	○	○

2. How satisfy you are with the amount of health information available about COVID-19?
   ○ Very satisfied
   ○ Satisfied
   ○ Dissatisfied
   ○ Very dissatisfied

◯ Don't know

3. Have you heard of the following…

	Heard	No heard/ Don't know
a. Number of cases infected by COVID-19	◯	◯
b. Number of deaths infected by COVID-19	◯	◯
c. Number of recovered cases infected by COVID-19	◯	◯

4. How do you mainly obtain health information?
◯ Internet
◯ Television
◯ Radio
◯ Newspaper
◯ Family members
◯ Other, please specify ___________

5. How confident are you in your own hospital to diagnose or recognize COVID-19?
◯ Very confident
◯ Somewhat confident
◯ Not very confident
◯ Not at all confident
◯ Don't know

6. Please rate your likelihood of …	Very likely	Somewhat likely	Not very likely	Not likely at all	Don't know
a. Contracting COVID-19 during the current outbreak	◯	◯	◯	◯	◯
b. Surviving COVID-19 if infected	◯	◯	◯	◯	◯

7. Please rate your concerns about other family members getting COVID-19.
◯ Don't have family member
◯ Very worried
◯ Somewhat worried
◯ Not very worried
◯ Not worried at all
◯ Don't know

8. Do you feel that you are being discriminated by other countries due to the outbreak of COVID-19?
◯ Yes
◯ No

◯Don’t know

9. Did you buy masks during the outbreak of COVID-19?

◯ Yes

◯ No

◯ No answer

10. When you bought a mask, was there something inconvenient.

please specify _________

Part E: Pre-cautionary measures in past 14 days

Do you do the following in the past 14 days…	Always	most of the time	sometime	occasional	Never
1.Covering mouth when coughing and sneezing	○	○	○	○	○
2.Avoid sharing utensils	○	○	○	○	○
3. Washing hands with soap and water	○	○	○	○	○
4. Washing hands immediately after coughing, rubbing nose or sneezing	○	○	○	○	○
5. Wearing mask regardless the presence or absence of symptoms	○	○	○	○	○
6. Washing hands after touching contaminated objects	○	○	○	○	○
7. Avoiding elevators	○	○	○	○	○
8. Sitting in one row while having a meal	○	○	○	○	○
9. Avoiding meeting more than 10 people	○	○	○	○	○

10. Do you feel that worry or anxiety has been made about COVID-19?

◯ Always

◯ Most of the time

◯ Sometime

◯ Occasional

◯ Never

11. How many extra hours per day do you stay at home to avoid COVID-19?

______ Hours

Part F Additional information about COVID-19

1. Would you like to receive additional information about COVID-19?

◯ Yes

◯ No (Skip to PHQ-9)

2. I would like to receive additional information about COVID-19 on …

		Yes	No
a.	Details on symptoms	◯	◯
b.	Advice on prevention	◯	◯
c.	Advice on treatment	◯	◯
d.	Regular updates for latest information	◯	◯
e.	Regular updates for the Outbreaks	◯	◯
f.	Advice for people who might need more tailored information, such as those with pre-existing illness	◯	◯
g.	Availability and effectiveness of medicine/vaccine	◯	◯
h.	How many people are affected/where it is affected	◯	◯
i.	Travel advice	◯	◯
j.	How COVID-19 is spread	◯	◯
k.	What other countries are doing	◯	◯
l.	The treatment and test cost of COVID-19	◯	◯
m.	The school precautions	◯	◯

3. Please specify other information you would like to receive about COVID-19

____

Part 2: PHQ-9

How often have they been bothered by the following over the past 2 weeks?

Question	Not at all	Several days	More than half the days	Almost every day
I seemed have no interest or pleasure in doing work or leisure activities.	0	1	2	3
I felt depressed and hopeless.	0	1	2	3
I found it difficult to fall asleep or keep sleeping, or slept too much suddenly.	0	1	2	3
I felt tired or had little energy.	0	1	2	3

I tended to have no appetite or overeat.	0	1	2	3
I felt that I have no confidence. Or I felt like I am a failure and let myself or my family down.	0	1	2	3
It was difficult to concentrate on such things as reading newspapers or watching television.	0	1	2	3
When someone was watching me, I felt like moving or talking slowly.On the contrary, It was more active than usual, too restless or impetuous	0	1	2	3
I thought that it is better to die or that I would harm myself anyway	0	1	2	3

Note: New PHQ-9 was included in the survey from 2017 in the community health survey.

0-4 points: no or minimal depression / 5-9 points: mild depression

10-14 or higher: depression present / 15-19: severe degree of depression / 20 or higher: depression is very severe

***In this study, depression is judged by prior research and clinical criteria and community health survey depression criteria if 10 or more points or higher.**
